# Quality assurance for automatically generated contours with additional deep learning

**DOI:** 10.1186/s13244-022-01276-7

**Published:** 2022-08-17

**Authors:** Lars Johannes Isaksson, Paul Summers, Abhir Bhalerao, Sara Gandini, Sara Raimondi, Matteo Pepa, Mattia Zaffaroni, Giulia Corrao, Giovanni Carlo Mazzola, Marco Rotondi, Giuliana Lo Presti, Zaharudin Haron, Sara Alessi, Paola Pricolo, Francesco Alessandro Mistretta, Stefano Luzzago, Federica Cattani, Gennaro Musi, Ottavio De Cobelli, Marta Cremonesi, Roberto Orecchia, Giulia Marvaso, Giuseppe Petralia, Barbara Alicja Jereczek-Fossa

**Affiliations:** 1 Division of Radiation Oncology, IEO European Institute of Oncology IRCCS, Milan, Italy; 2 Division of Radiology, IEO European Institute of Oncology IRCCS, Milan, Italy; 3 Department of Computer Science, University of Warwick, Coventry, Warwick, CV4 7AL UK; 4 Molecular and Pharmaco-Epidemiology Unit, Department of Experimental Oncology, IEO European Institute of Oncology IRCCS, Milan, Italy; 5 Department of Oncology and Hemato-Oncology, University of Milan, Milan, Italy; 6 Radiology Department, National Cancer Institute, Putrajaya, Malaysia; 7 Division of Urology, IEO European Institute of Oncology IRCCS, Milan, Italy; 8 Medical Physics Unit, IEO European Institute of Oncology IRCCS, Milan, Italy; 9 Radiation Research Unit, IEO European Institute of Oncology IRCCS, Milan, Italy; 10 Scientific Direction, IEO European Institute of Oncology IRCCS, Milan, Italy; 11 Precision Imaging and Research Unit, Department of Medical Imaging and Radiation Sciences, IEO European Institute of Oncology IRCCS, Milan, Italy

**Keywords:** Quality assurance (Health care), Confidence calibration, Diagnostic imaging, Prostate, Magnetic resonance imaging

## Abstract

**Objective:**

Deploying an automatic segmentation model in practice should require rigorous quality assurance (QA) and continuous monitoring of the model’s use and performance, particularly in high-stakes scenarios such as healthcare. Currently, however, tools to assist with QA for such models are not available to AI researchers. In this work, we build a deep learning model that estimates the quality of automatically generated contours.

**Methods:**

The model was trained to predict the segmentation quality by outputting an estimate of the Dice similarity coefficient given an image contour pair as input. Our dataset contained 60 axial T2-weighted MRI images of prostates with ground truth segmentations along with 80 automatically generated segmentation masks. The model we used was a 3D version of the EfficientDet architecture with a custom regression head. For validation, we used a fivefold cross-validation. To counteract the limitation of the small dataset, we used an extensive data augmentation scheme capable of producing virtually infinite training samples from a single ground truth label mask. In addition, we compared the results against a baseline model that only uses clinical variables for its predictions.

**Results:**

Our model achieved a mean absolute error of 0*.*020 ± 0*.*026 (2.2% mean percentage error) in estimating the Dice score, with a rank correlation of 0.42. Furthermore, the model managed to correctly identify incorrect segmentations (defined in terms of acceptable/unacceptable) 99.6% of the time.

**Conclusion:**

We believe that the trained model can be used alongside automatic segmentation tools to ensure quality and thus allow intervention to prevent undesired segmentation behavior.

## Key points

- A deep learning model is trained to predict segmentation quality.
- Data augmentation provides a means to expand the data set almost infinitely.
- The model achieves 99.6% accuracy and 2.2% mean percentage error.
- The model can be used to assure a desired standard is met.

## Introduction

Segmentation of anatomical structures in medical images is a vital step in many clinical domains including radiology, pathology, ophthalmology, dermatology, and microscopy [1–4]. For instance, accurate delineations of neighboring organs are crucial for calculating dose and assessing risks in radiotherapeutic treatment planning [5]. Most contemporary research in medical image segmentation focus on developing and applying automatic segmentation procedures, primarily with deep learning (DL) models, to reduce the workload of clinicians, speed up the delineation process, and improve the segmentation quality. As the performance of these models has improved, institutions are looking to start experimenting with them in clinical practice. During research focused on developing and validating the models, however, several aspects of model deployment have been left unaddressed, including model drift, underspecification (that is, when pipelines can return many predictors with equivalent training performance, but with very different deployment performance) [6], dataset and model biases, and quality assurance. Indeed, the authors of a recent survey of AI in radiation oncology [5] argued that there is an unmet need for guidance on the implementation and use of AI models in clinical practice.

Quality assurance (QA) is a key step in the deployment of any AI algorithm or model [5, 7–10]. In general, it refers to the practice of monitoring the output, performance, and user experience of a deployed method or model to ensure that it is working as intended. This is of particular importance in medical contexts, where patient outcomes may be jeopardized. Previously, the role of humans in this step has been mostly subsumed, but there appears to be no principled reason why this cannot be carried out by AI algorithms as well. Despite the potential benefit of AI and machine learning (ML) for QA being recognized [11], there is surprisingly little literature on the topic, particularly in the field of image segmentation.

In ordinary classification tasks, the output of the model is typically a vector of probabilities that represents the model’s certainty for a given sample. This vector can be used to give users a measure of how confident the model is for a given prediction (although the reliability of this confidence estimation is debated—see the so-called calibration problem). For segmentation models, on the other hand, the output is typically a pixel-wise collection of probabilities, which makes its direct use ambiguous. One way to overcome this is to use an ensemble of multiple segmentation models to build an uncertainty prediction model based upon the variance of their predictions, as suggested in [12]. However, ensembling multiple models require drastically more time and resources, particularly in the case of DL. Another approach is to use a Bayesian framework, which inherently models uncertainty [13]. Men et al. [14] tried to solve the problem by studying a binary definition of quality (e.g., errors are present/absent), while other researchers have suggested using rule-based models derived from geometric attributes [15, 16], or texture-based [17], volumetric-based [18], and/or shape- and intensity-based [19] features with ML and statistical models. While these methods can improve the quality evaluation of contours, they suffer from the inherent limitations of handcrafted models, which are often coarse-grained and typically produce inferior results. In this context, DL models, whose superior feature extraction and inference capabilities have been demonstrated in a multitude of other image analysis domains, may provide invaluable advantages.

Research relating to QA of segmentation models with DL is very limited. Chen et al. [20] conducted a QA study on 680 breast cancer patient CT images based on a ResNet-101. Their approach achieved good performance but used a discrete classification rather than a continuous regression method, which could potentially be a limitation in clinical contexts if flexibility is desired (since the classification threshold cannot be changed dynamically). Two other related approaches are the fields of uncertainty prediction and out-of-bounds detection. In uncertainty prediction problems, the task is to estimate the uncertainty of the network given an input prediction pair. (This is typically studied in regression problems where network outputs are not represented as certainties.) Traditional approaches in this field model the uncertainty with a Bayesian framework and typically require either explicit models of the ground truth distribution [21–23] or joint training with an uncertainty network [24]. While joint training can be an effective and well-versed approach, it stands to reason that it cannot be implemented after training. Instead, out-of-bounds detection simply tries to detect samples that differ greatly from the training distribution. These detectors often rely on artificially created out-of-distribution samples (possibly with a generator neural network [25]) [26], which is a difficult problem in itself that introduces another dimension of bias. Due to the scarcity of research directly related to QA, it can be useful to draw inspiration from other domains that predict (continuous) outcomes directly from images. One example is age estimation, where the task is to predict a person’s age from pictures of their face (see, e.g., [27–30]). Notably, all state-of-the-art models in this field are DL architectures. However, a key thing that distinguishes segmentation QA from age estimation is the presumed in-sample interaction between images and contours: it is impossible to tell the quality of a segmentation just by purely looking at the segmentation (or image).

There are three main ways to predict the performance of automatically generated contours: regressing the performance metric directly, predicting some discrete or qualitative measure of performance (e.g., good/moderate/bad), or predicting the ranking of samples ordered by quality (i.e., ordinal regression/classification). The second method is useful when precise ground truth segmentations are not available, or when time constraints limit the annotation quality of the training data since clinicians can allocate samples to qualitative categories faster and more easily than they can produce reliable ground truth segmentations. The third method can be beneficial when only the ordering of samples is important, but the downside is that single-sample inference is not straightforward, particularly for out-of-distribution samples. We opted for the first method, as it is good when many training samples are available, preferably over a wide distribution of ground truth segmentation scores. It allows for the use of distance-based loss metrics, which penalize poor predictions more than good ones. In this work, we sought to:

- Build a convolutional DL model to predict the quality of automatically generated contours. This model can be used in QA to give automatic segmentation models a greater sense of transparency.
- Compare the model to a naïve baseline that predicts the segmentation performance from patient characteristics only. If poor performance can be estimated from clinical variables alone, clinicians may be able to leverage this information to exclude subsets of patients on which automatic segmentation methods produce poor results.
- Investigate when the model is able to correctly handle likely failure cases with noisy, empty, or misaligned predictions. Identifying failure cases is a crucial step in QA and analyzing this behavior may provide insights into potential model biases and model behaviors.

## Methods

### Dataset

To train the quality prediction models, we used a set of 60 prostate MRI images along with automatically generated contours that had previously been produced by a bespoke deep segmentation network [38, 39] with a modified 3D-adopted EfficientDetB0 [36] architecture (see Sect. 2.4 for additional details on this architecture). Each image contour pair had an accompanying ground truth segmentation mask against which the quality of the automatically generated contours could be calculated. The ground truth segmentations were generated manually by consensus from two expert radiologists (> 5 years’ experience). In addition, 20 images had an additional set of automatically generated contours produced by a slightly different network, such that the model could learn to distinguish the difference between different contours on the same image. In total, there were 60 prostate images and 80 automatically generated contours. The Dice values of the contours were all in the [0.847, 0.943] range.

The images used for segmentation were axial T2-weighted MRI scans of the prostate acquired using a 1.5 T scanner (slice thickness 3.0–3.6 mm, slice gap 0.3 mm, pixel spacing 0.59 × 0.59 mm, echo time 118 ms, and repetition time 3780 ms) [31, 32]. All images underwent N4 bias field correction (SimpleITK 2.0.2 with default parameters) before segmentation and model training, and all images had equal voxel sizes.

The following clinical characteristics were available for each patient for use in the baseline model: age, prostate volume, ISUP grade, PI-RADS score, iPSA, and risk class.

The study was performed within the notification presented to the Ethics Committee of IRCCS Istituto Europeo di Oncologia and Centro Cardiologico Monzino (via Ripamonti 435, 20,141 Milano, Italy) (CE notification n. UID 2438). All patients had given their consent for use of their data for research and educational purposes.

### Predicting contour quality

**Fig. 1:**
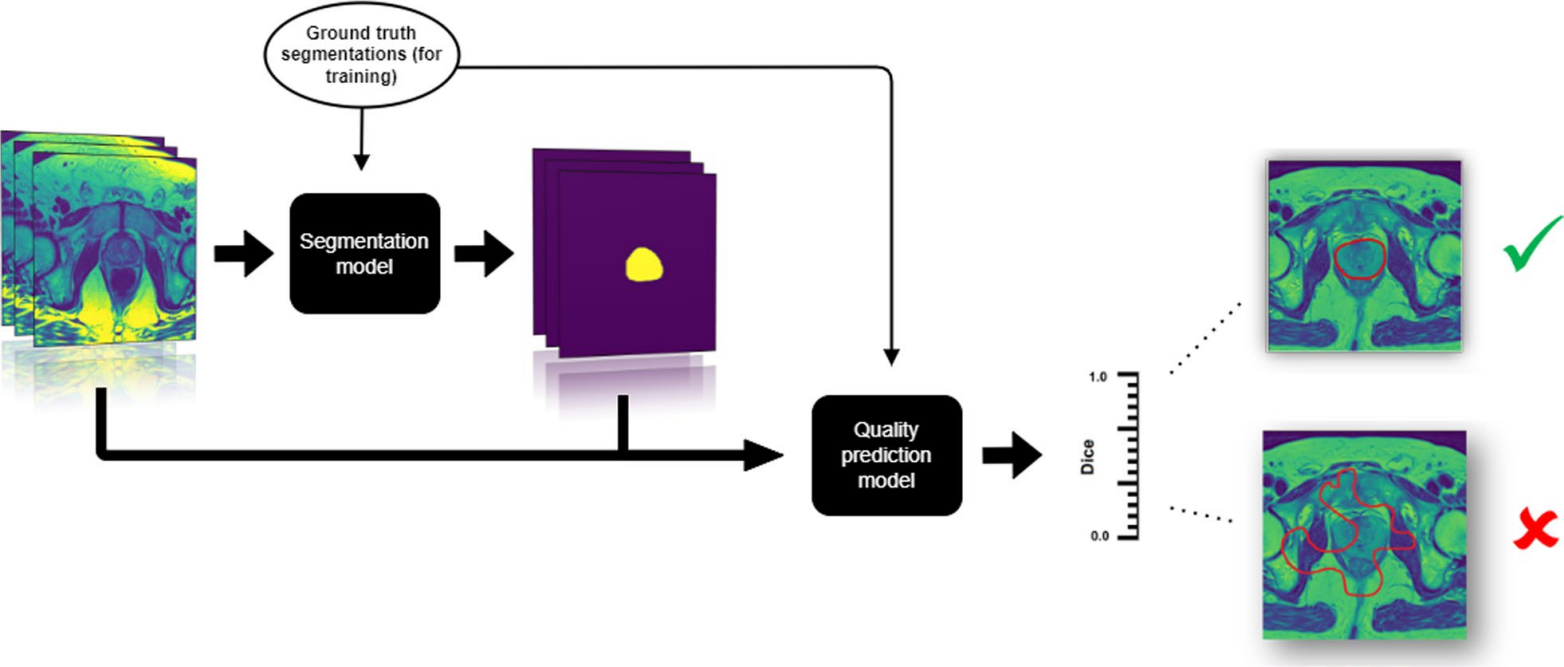
Overview of the problem of predicting the quality of organ segmentations. First, a segmentation model (not covered in this article) takes images as input and produces segmentation maps. Then, our quality prediction model takes both the images and the segmentation maps as input and produces an estimate of the quality of the segmentation—in this case the Dice similarity coefficient. Good contours have a high Dice value and poor contours have a low Dice value. Note that we need ground truth segmentations in order to calculate the true Dice value and train the quality prediction model with supervised learning. In this study, we used 60 ground truth segmentations and 80 automatically generated masks along with heavy data augmentation to train the quality prediction model

We framed the problem of quality assurance as a regression problem, where the input to the model is an image contour pair and the output is a measure of quality (see Fig. 1 for an illustrative overview). The specific quality metric we chose was the Dice coefficient because it is mathematically well defined, easily interpreted, bounded, and widely used within the medical imaging community.

For two binary pixel arrays (e.g., segmentation maps) A and B, the Dice coefficient is defined as

1 $${\textit{Dice}}\left( {A,B} \right) = \frac{{2\left| {A \cap B} \right|}}{\left| A \right| + \left| B \right|}$$

Its value ranges from 0 to 1 where 1 corresponds to perfectly overlapping segmentations and 0 corresponds to having no intersection.

To measure the performance of the quality prediction models, we used mean absolute error (MAE) between the predicted Dice and the target Dice values, as well as the Spearman rank correlation between them. The Spearman correlation measures how correct the order of an ordered set is, and ranges from 1 (all samples are placed in correct order) to − 1 (all samples are placed in the opposite order). Random placement has an expected rank correlation of zero. As such, it gives an intuitive understanding of how well the algorithm can tell good contours from bad ones. In this work, the use of rank correlation will be implied whenever correlations are mentioned.

### Baseline quality prediction model

The baseline model tries to predict how well an arbitrary segmentation algorithm would perform on a patient given only the clinical variables for that patient. The rationale for this is to examine whether any clinical variables are predictive of how hard it is to segment a given prostate. Because this model makes no use of images, it cannot distinguish between different segmentations of the same patient, but it can still be useful as an analytical tool and a benchmark. As the baseline model architecture, we chose a gradient boosted decision tree model implemented in CatBoost version 1.0.3 [33] with Python 3.7. For the 20 images with two different segmentations, we used the mean value of the Dice coefficients as the target value.

To train this model, we first perform a 64-step parameter search with the Optuna Python package [34] with default settings to find suitable parameters. The search space is displayed in Table 1. Each parameter set was evaluated by its mean absolute error after eight repeated random fivefold cross-validations. The best model was then further evaluated with 64 repeated random fivefold cross-validations.

**Table 1:** Parameter space searched by the Optuna parameter search for the baseline CatBoost model

Parameter	Values
n estimators	{1, 256}
max depth	{1, 6}
l2 leaf reg	[10^−3^*,*10]*
random strength	[0.1, 3]

^*^log-uniform prior

[]: continuous interval

{}: integer interval

In order to gauge the usefulness of the baseline model, we compared its performance against a naïve baseline that predicts the mean Dice value for all samples.

### Quality prediction network

**Fig. 2:**
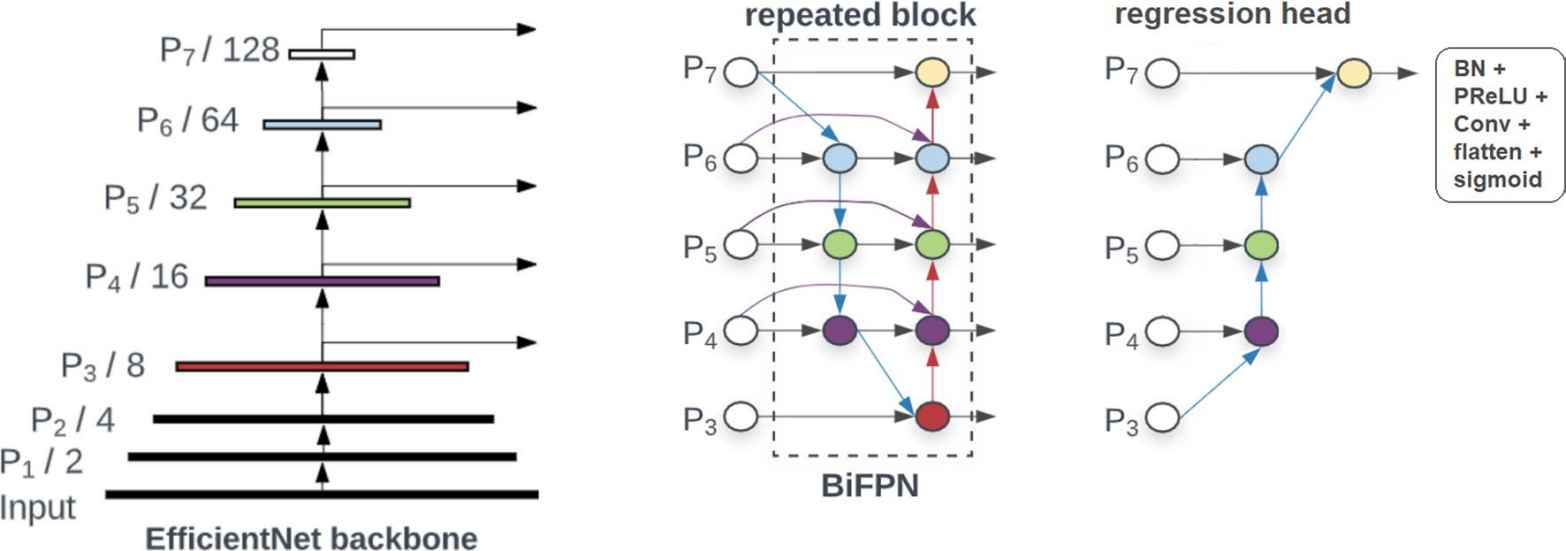
Network architecture. An EfficientNet B0 backbone is connected to three repeated bidirectional feature pyramid blocks (BiFPNs). The regression head consists of serially connected fast normalized fusion nodes and finally BatchNorm (BN), PReLU, a single-channel convolution, and a sigmoid activation. Numbers indicate the resolution at each level relative to the input. Image adapted from [35]

**Fig. 3:**
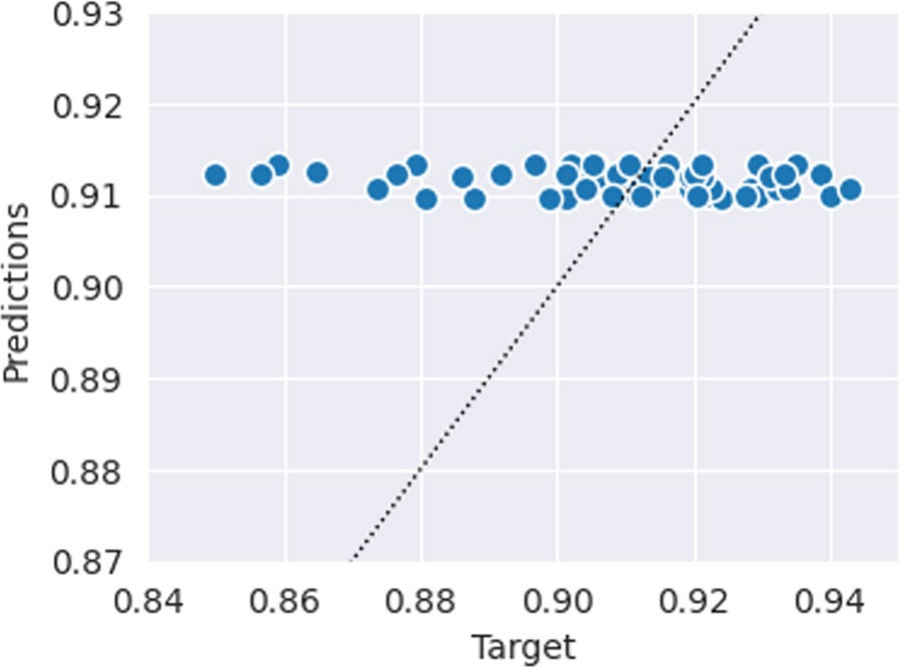
Predicted vs. target Dice values of the baseline CatBoost model, which only uses clinical variables to predict segmentation quality. The dotted line indicates perfect *x* = *y* predictions. The predictions of this model tend to only vary minimally (very close to the naïve model), suggesting that the clinical variables are not indicative of segmentation performance

The deep learning model we trained to predict segmentation quality was a modified EfficientDet [35] architecture (see Fig. 2). This architecture is an extension of the EfficientNet model [36] that is tailored toward object detection—it includes an EfficientNet backbone with seven levels (P1 to P7) connected to repeated “BiFPN” blocks (Bidirectional Feature Pyramid blocks). Our modifications included adaptation to 3D convolutions as well as an expansion factor reduction (from 6 to 2) and a custom regression head. The regression head consisted of serially connected fast normalized fusion nodes (see [35] for details) followed by batch normalization, PReLU, a single-channel convolution, and a final sigmoid activation function. The EfficientNet [36] backbone was a B0 type with the default filter parameters of 32, 16, 24, 40, 80, 112, and 192 channels for the P1 to P7 levels, respectively. Our BiFPN blocks were repeated three times and used 64 filters each (Fig. 3).

To reduce the memory consumption of the model, the images were center-cropped from 320 × 320 × 28 to 160 × 160 × 28 voxels. The images were also normalized by linearly mapping the 0th and 99th percentiles to the [0, 255] range, after which the 100th percentile values were appended. The MRI images and segmentation maps were concatenated on the channel dimension to form 4D tensors of shape 160 × 160 × 28 × 2 for each sample. These 4D tensors were used as the input to the model.

The network was trained for 200 epochs with MSE loss and batch size 2. We used the Adam optimizer with a learning rate of 0.002, which was reduced to 0.0002 after 120 epochs. Validation of the model was done with a random fivefold cross-validation.

To give the network the ability to interpolate outside the narrow range of target Dice values typical of prostate segmentation, we used an elaborate data augmentation scheme to generate novel contours. At each epoch, one of the two samples had its corresponding contour switched with another contour randomly chosen from the training set such that each batch consisted of one real and one “fake” image contour pair. The fake contour was then scaled by a random factor in [0.55, 1.8]. After this procedure, we also applied standard data augmentation two both samples (in order): horizontal flips, uniform in-plane rotation (in the $\pm \frac{\pi }{12}$ range), uniform 2D *x* and *y* translation (in  ± 10%), uniform zoom (in  ± 10%), and elastic deformation. This procedure also eliminates bias that could be introduced by only using contours from a single segmentation model.

### Failure case studies

We evaluated how well the model predicts the quality of different variations of failed contours, for which the predicted Dice score ought to be low. The following failure modes were investigated (see Fig. 7 for illustrations):

1. empty contours (every pixel in the array is zero—no prostate tissue has been identified),
2. uniform binary noise (each pixel in the array is randomly assigned a value of zero or one),
3. filled matrix of ones (every pixel in the array is one—the whole image has been identified as prostate tissue)
4. shifted ground truth masks (the ground truth segmentation is randomly shifted uniformly by ± 50% in the *x-* and *y-*direction).

These cases were constructed from the 16 patients in the test set at each validation fold, such that each failure case generated 80 independent samples in the course of the cross-validation procedure.

In addition, we evaluated the predictions on the 16 unseen ground truth segmentations at each validation fold (for a total of 80 ground truth images). This allowed us to test the model performance on the opposite end of the domain, where all target values are 1.

We also defined a global accuracy score to indicate how well the model performed across all test samples (80 from the standard test set, 320 failure case samples, and 80 ground truth samples). This is useful because the MAE is not always indicative of how helpful the model’s predictions are. For example, if there is a segmentation with a true Dice value of 0.0, and the model predicts a Dice value of 0.5, the contour would still be flagged as “poor quality,” because both 0.0 and 0.5 Dice are considered bad. This means that the prediction is *qualitatively* correct, even though the MAE of 0.5 is very large. For a predicted Dice value *ŷ* and target Dice value *y*, we defined a failed prediction as either:

1. (*ŷ* < 0*.*75)∧(*y* > 0*.*8), i.e., a predicted Dice value of less than 0.75 when the target Dice value is larger than 0.8, or
2. (*ŷ* > 0*.*8)∧(*y* < 0*.*75), i.e., a predicted Dice value larger than 0.8 when the target Dice value is less than 0.75.

## Results

### Baseline model

**Fig. 4:**
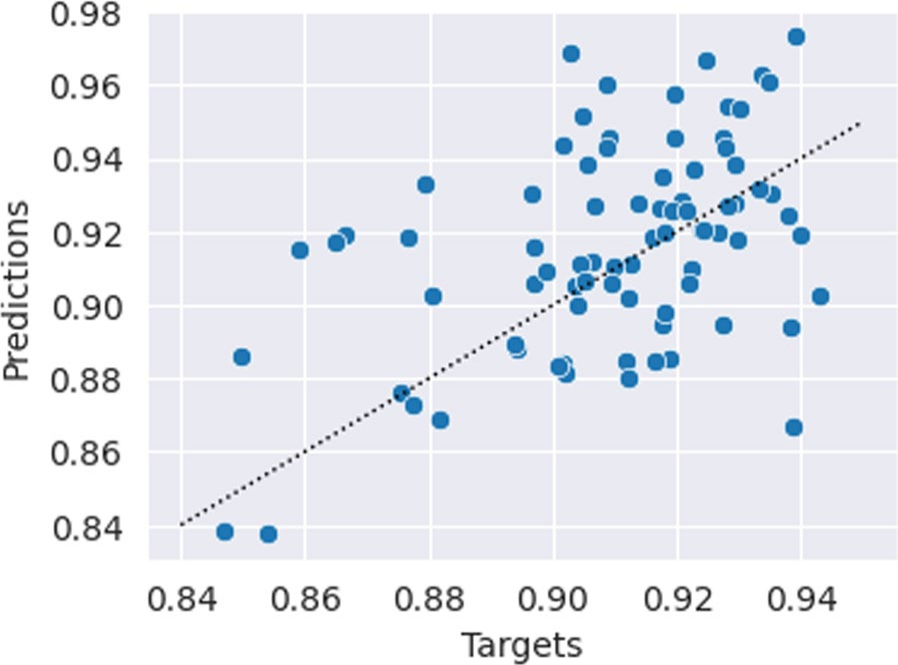
Predicted vs. target Dice values of the deep network shown in Fig. 2. The dotted line indicates perfect *x* = *y* predictions

The predicted Dice values by the baseline CatBoost model are shown in Fig. 3 and the performance of the CatBoost and naïve baseline models are summarized in Table 2. The MAE were 0.016 for both models, and the prediction–target correlation for the CatBoost model was − 0.155. The most important features in the CatBoost model were iPSA (68%) and volume (21%) (Fig. 4).

**Table 2:** Average mean absolute error (MAE) and rank correlation of the baseline CatBoost and naïve models, which only utilize clinical variables to predict segmentation quality. The naïve method predicts the mean target value for all samples. Parentheses indicate standard deviation. The values are aggregated from 64 repeated fivefold cross-validations

	MAE	Corr
CatBoost	0.016 (± 3·10^−4^)	− 0.16 (± 0*.*02)
Naïve	0.016 (± 0)	n/a

### Quality prediction network

**Fig. 5:**
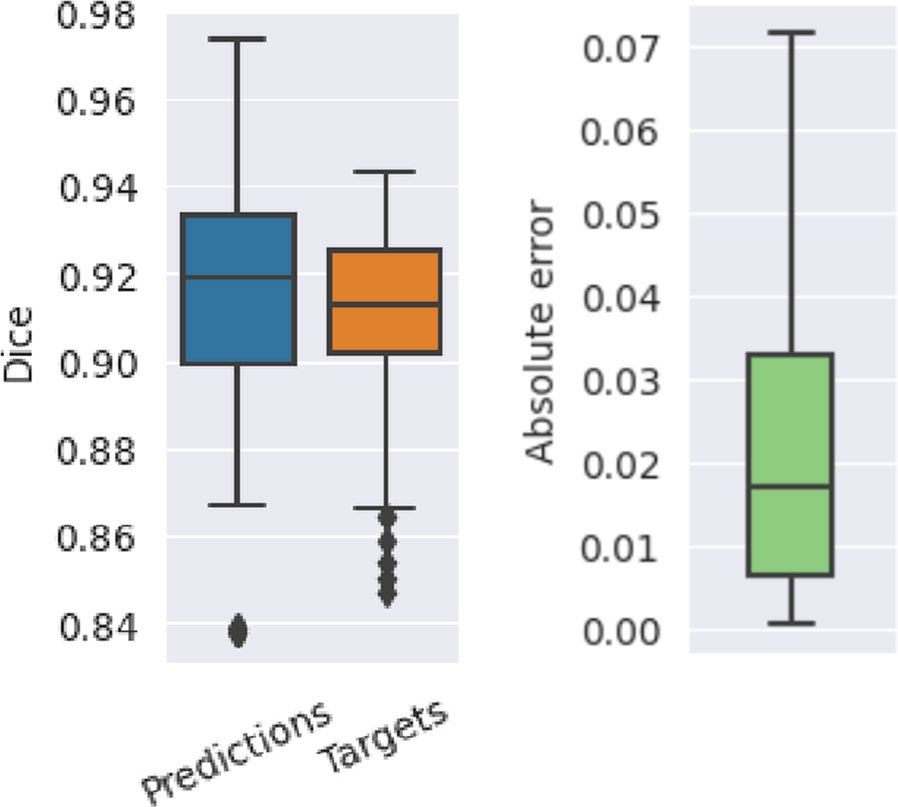
Predicted Dice values, targets, and the respective absolute error of the quality prediction deep learning network. The mean absolute error is 0.02 and the correlation between the predicted and target values are 0.42

**Fig. 6:**
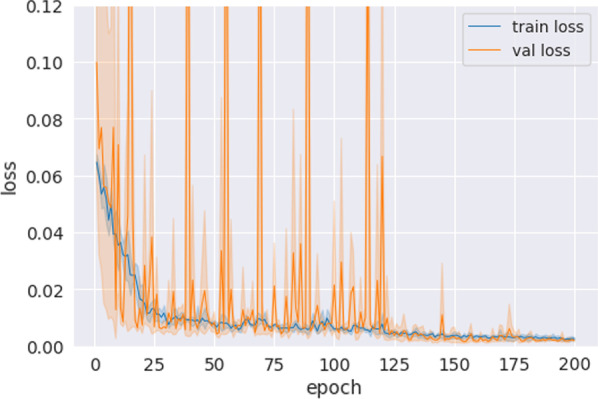
Characteristic training curves of the deep quality prediction network. The plot is an aggregate of all the different validation folds. The validation loss often spikes in early training, which then disappear safter the learning rate reduction at 120 epochs

The mean absolute error of the Dice value predictions was 0.020 ± 0*.*026 (2.2% mean absolute percentage error), and their correlation with the target values was 0.423 (Figs. 5 and 6). The maximum absolute error was 0.066 (equivalent to a 7.3% deviation from the target). The time required to generate predictions was 0.02 s per patient on an RTX 3090 GPU. Characteristic training curves of the network are shown in Fig. 4.

### Failure case studies

**Fig. 7:**
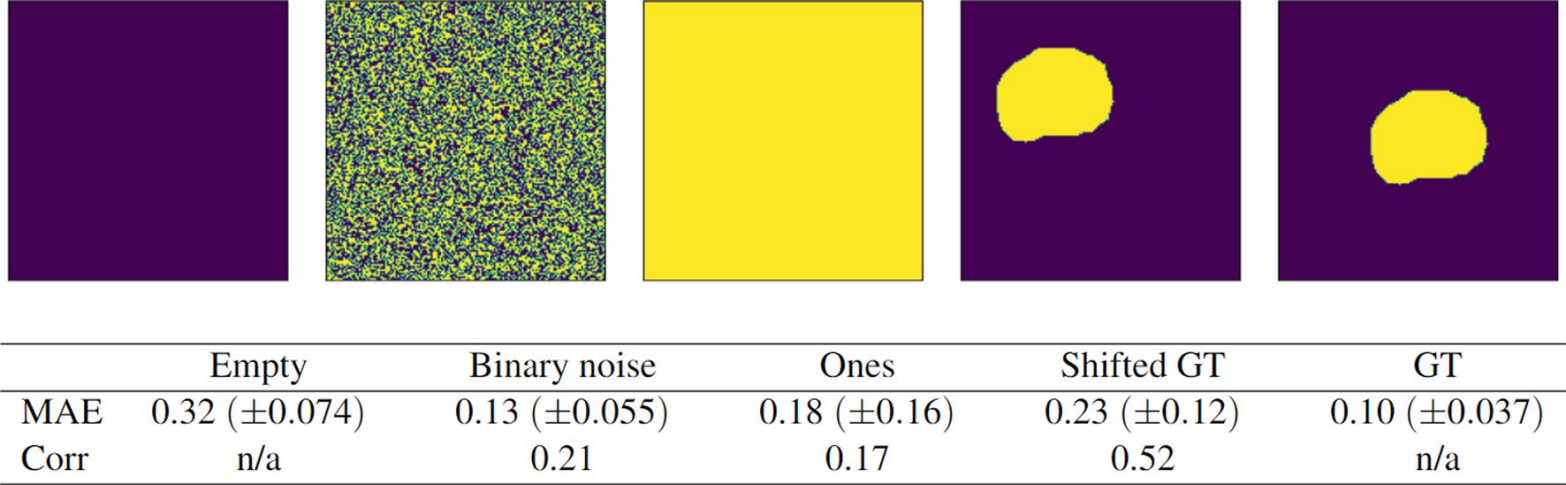
Performance of the quality prediction model (MAE and rank correlation of predicted Dice scores) on different cases of failed segmentations: completely empty contours, pure noise, matrices full of ones, and shifted ground truths (GTs). The performance on real GT segmentation maps is also shown. All results are aggregated over 5 different validation splits for a total of 80 samples each

The results of the predictions on the constructed failure cases are shown in Fig. 7 together with an example segmentation from each type of failure. The least successful cases were the empty contours (0.317 MAE), followed by the shifted GT segmentations (0.233 MAE), the all-ones segmentations (0.182 MAE), and the binary noise (0.126 MAE). The shifted GT cases, which had the second-worst MAE, had the best correlation with the target values: 0.522. The empty and GT contours have undefined correlations since their target values are all zeros and all ones, respectively.

In terms of overall accuracy, only two out of the 480 cases were misclassified, amounting to a 99.6% accuracy. The first case was a segmentation full of ones with a predicted Dice value of 0.81 and target Dice value of 0.11, and the second case was a shifted GT mask with a predicted Dice value of 0.82 and target Dice value of 0.72.

## Unsuccessful experiments

As well as the above experimental results, we also provide observations on some of the groundwork testing carried out in preparation for this work.

One architecture we tested used a confidence branch, which is an extension that can be made to arbitrary networks [37]. The confidence branch is trained to output an estimate of how confident the network is in its predictions. This can be achieved by letting the network “peak” at the correct answers (with a penalty) during training in order to output more correct answers. The intuition behind this is that peaking is only profitable for inherently uncertain predictions. We did not manage to get this branch to output useful values—the confidence always converged to either 0 or 1 for all samples, even after introducing a budget parameter.

Another DL network we experimented with used a regression head added directly to the EfficientNet backbone, which requires much less memory and training time. This worked well in terms of training MSE but was incredibly noisy. A similar scenario occurred when we trained our final network architecture with fewer BiFPN layers and/or filters.

## Discussion

Our DL quality prediction model accurately predicted the Dice score of automatically generated prostate contours with a MAE of 0.020. This amounts to a mean deviation from the true Dice values of only 2.2%. In particular, none of the errors were larger than 0.066 (7.3% deviation from the target), which indicates a high degree of robustness and reliability. The moderate correlation of 0.42 between the predicted and target values suggests that the model is also able to correctly tell qualitative differences (i.e., which ones are better/worse) between contours, even when the differences are minor.

The performance in the failure cases might seem alarming when looking at their MAE values, which range from 0.126 MAE on binary noise to 0.317 on empty contours. However, this would likely not be a major problem in practice since the overall accuracy of the model was 99.6%. For example, a large MAE does not necessarily indicate a failure of the model when both the target and predicted Dice values are low. Our model was not trained on any contours with target Dice values of 0.0, and as such had no way to interpolate to this regime. Anticipating failure cases and including such cases in the training set is one way to boost the model’s reliability. Furthermore, a well-built segmentation model being deployed in practice ought to not output obviously poor segmentations (assuming no model drift), and such failures are easy to spot by simple inspection without the need for an external quality assurance model.

The naïve model that predicts the mean of the target Dice values for every patient achieved a MAE of 0.016, which is lower than the DL model’s MAE of 0.02. However, the naïve model would not be able to identify failure cases and qualitative differences between contours, since all its predictions are identical. Similarly, our baseline CatBoost model, which only used clinical characteristics to predict the quality of automatically generated contours, also had a MAE of 0.016. The low variance along with the negative correlation of the CatBoost predictions (Fig. 3 and Table 2) suggests that this model has no merit over the naïve model, effectively rendering it useless in practice. This indicates that the performance of automatic segmentation models cannot be inferred from clinical characteristics alone. This should not be too surprising given that the model is not able to distinguish different segmentations on the same patient.

An obvious question to raise is: If we need a deep network to safeguard the performance of the segmentation network, should we then not need a deep network to safeguard the performance of the safeguard network? The predicament is that, if the performance of the first network could be guaranteed, we would not need a safeguard network in the first place, and if not, we would potentially need an infinite chain of networks. It is likely, however, that the utility of such networks diminishes the further down the chain you go because the error is necessarily reduced by a nonzero amount each step. An analogy can be drawn to gradient boosted machines, which are chains of prediction models trained on a propagated error signal. These models are usually trained with decision trees because decision trees are extremely fast to train. On the other hand, for DL models in computer vision where training times often exceed hours or even days, it should be clear that using more than a few chained safeguard networks is practically infeasible.

While similar studies we found focused on either binary error detection or discretized ordinal regression, our model performs continuous regression. In general, this should be preferred, since it is more general and often enables better performance. This approach also enables a dynamic definition of error detection that can be changed on the fly, which may be valuable in a medical context. The only other study we found that regresses Dice scores directly achieved a MAE of 0.06 [20] on breast cancer segmentation, which is three times higher than our MAE of 0.020.

One thing to note is that, if the Dice prediction network is accurate enough, the original segmentation network could utilize the predicted Dice values to inform its own gradients, for instance by using the Dice prediction network as a discriminator in a GAN training scheme. This is a potential direction for future research but was out of scope for this study.

While this article demonstrates a novel way to assess the quality of automatically generated contours, it is important to note that external and rigorous validation is needed before the methods can be reliably applied in practice. With the limited dataset of 80 patients, the results herein can only be considered as a proof of concept. Ideally, a test set of unseen data should be used to assess real out-of-sample performance and reproducibility, but we opted to not use a held-out set in order to give the training and validation sets more coverage. Too small datasets risk being overly dominated by random effects, which can compromise the learned representations and the performance estimation. At present, there is no universally accepted solution to these tradeoffs, especially in small data scenarios. It can also be discussed whether it is wise to use an AI algorithm to assess the output of another AI algorithm in clinical practice, and when human supervision should be requested. At the very least, it seems natural to demand human supervision in early applications of AI in healthcare. One benefit of QA models like ours is that they can easily be deployed alongside a human professional in order to alleviate the workload and improve his/her judgment.

## Conclusions

In this work, we trained a deep learning model to predict the quality of prostate contours in terms of their Dice similarity coefficient with the ground truth labels. The model can be used in practice to ensure quality and monitor the performance of deployed automated contouring models. Our results showed an absolute deviation from the target values of only 0.016, which is equivalent to a 1.7% deviation. With suitable retraining, the model could also be applied to any other segmentations.

## Data Availability

The datasets generated and/or analyzed during the current study are not publicly available due to privacy concerns but are available from the corresponding author on reasonable request.

## Abbreviations

DL: Deep learning
GT: Ground truth
MAE: Mean absolute error
ML: Machine learning
MRI: Magnetic resonance imaging
MSE: Mean squared error
QA: Quality assurance
